# Saved by Design? The Case of Legal Protection by Design

**DOI:** 10.1007/s11569-017-0299-0

**Published:** 2017-08-25

**Authors:** Mireille Hildebrandt

**Affiliations:** 1 Vrije Universiteit Brussel, Brussels, Belgium; 2 Radboud University, Nijmegen, Netherlands

**Keywords:** Legal protection by design, Affordance, Capability, Data-driven architectures, Rule of Law, Natural language, Computational infrastructures

## Abstract

This discussion note does three things: (1) it explains the notion of ‘legal protection by design’ in relation to data-driven infrastructures that form the backbone of our new ‘onli*f*e world’, (2) it explains how the notion of ‘by design’ relates to the relational nature of what an environment affords its inhabitants, referring to the work of James Gibson, and (3) it explains how this affects our understanding of human capabilities in relation to the affordances of changing environments. Finally, this brief note argues that ‘safer by design’ in the case of nanotechnology will require legal protection by design to make sure that human capabilities are reinvented and sustained in nano-technical environments.

## Introduction

This brief discussion note enquires into ‘legal protection by design’ (LPbD), an idea that is similar to but not identical with the subject of this special issue: ‘safer by design’. LPbD emerged on the cusp of data science and law, raising the pivotal question of whether we can be saved by design when written law fails to save us from the pitfalls of data-driven architectures. Since this question is also relevant for the notion of ‘safer by design’ in the case of nanotechnology, I share my thoughts on the issue. Before introducing LPbD, let me first clarify that I will refer to ‘design’ as the process of developing and engineering specific technologies, as well as the process of introducing and employing them in human society (design as a verb), including also the result of that process (design as a noun). Design should not be understood as merely a matter of shaping the interface; it also concerns the back end of technological systems and the way they frame interactions on the front end. Second, ‘by design’ is not a panacea. Rather, it may be a precondition that enables safe, fair, accountable and transparent research and employment of data-driven architectures, nanotechnology and other new technologies. Whether we manage to achieve such laudable ends will depend on other factors, but if we neglect ‘safer by design’ as a first principle, we are in for trouble. Third, to instigate ‘safer by design’, we will need more than ethics. I will therefore conclude that ‘safer by design’ will be dependent on LPbD for its success as a precondition, as this turns ‘safer by design’ into a legal obligation for all those involved in researching, developing, marketing and providing products involving nanotechnology.

## The Need for Legal Protection by Design in the onli*f*e World

The idea of LPbD has arisen from the mutation of law’s environment, which has moved *from language to computation* when it comes to the framing of reality. Today, much of the framing is done or prepared by data-driven machines that feed on our behavioural data. Au fond, these machines present humans with a choice architecture that is based on automated AB testing and predictive or even pre-emptive machine learning contraptions. To describe the life world that is produced by the combination of big data, learning algorithms and myriad cyber-physical infrastructures, some have proposed to use the notion of an onli*f*e world [1]. This refers to the transformation of offline environments into online realms, exemplified by the Internet of things, self-driving cars, smart energy grids and other cyber-physical infrastructures. The onli*f*e world also refers to the fact that the combination of computational back end systems, hyperconnectivity and machine learning basically turns our inorganic environment into an animated world, ‘populated’ by *mindless* interacting machines that anticipate our behaviours and act upon the predictions they generate. The latter implies that these architectures do not merely turn everything online; due to machine learning and machine agency, *things* also—seemingly—*come alive*. Even though they have no consciousness, let alone self-consciousness, they foresee us and act on that. Finally, the notion of an onli*f*e world suggests that the online realm has penetrated our life world; it has become our new ‘everyday’, the air we breathe. It informs how we experience our own life.

My assumption here is that the *choice architecture* of everyday life is increasingly *designed* or *engineered* instead of *given*, constructed by complex computational systems upon which we become ever more dependent. This implies that central tenets of the law, such as individual consent, freedom of information, fairness and legal certainty can no longer be taken for granted. The ‘existence’ of these legal tenets has been dependent upon the decisions of legislators and courts that produce legal code and case law, both of which attribute legal effect if specific legal conditions apply. Such decisions and the ensuing legal effect have thus been contingent upon what is afforded by the *technologies of the word*, [2] notably the print-wise multiplication of natural language text. The shift from natural language to computation as the most influential means to frame reality implies—as I have argued elsewhere—that the *mode of existence* of law-as-we-know-it is on the verge of changing beyond recognition [3, 4]. If the law is meant to regulate a world generated by computational systems, it must learn to speak the language of computation. The need for LPbD is not, therefore, based on the desire to enforce legal compliance (as with ‘legal by design’, ‘computational law’ or ‘techno-regulation’), [5] but—on the contrary—based on the awareness that current technological ecosystems are disrupting the meaning and the substance of *legal effect* and the *force of law* [6],^1^ notably in the realm of the protection of fundamental rights.

Computing systems, by now, mould the choice architecture of human interaction to an extent that uproots previous ways of ordering that were based on what sociologists like to call ‘institutions’. The sociological notion of an institution refers to consolidated patterns of human interaction shaped by mutual expectations. The ensuing web of anticipations that informs interacting individuals grounds institutions such as marriage, employment, religious worship, the market and education. This grounding is accomplished by means of natural language. Ηuman language facilitates shared meaning and ‘common sense’, as well as the ability to disrupt such meaning by means of creative resistance or simply by generating successful misunderstandings that—in turn—lead to subtle or not so subtle shifts in meaning.^2^ Institutions create stability in the face of these iterant shifts in meaning that in turn feed on the inherent ambiguity of the human language. This ambiguity is both unsettling and productive, creating both uncertainty and possibility. Computing systems, however, speak another language, whether they are deterministic (as with block-chain) or somewhat unpredictable (as with machine learning). Nevertheless, just like institutions used to do, networked computer systems often determine the options for their end-users, while hiding the myriad operations in their so-called backend, to provide a smooth user experience or to nudge end-users in the direction that fits whoever developed (or paid for) the system. Whereas the ambiguity of natural language enables the contestation of received opinion as well as expert evidence, precisely because it builds on a semantics that is always on the move, this is not evident in the case of computer language. The latter requires unambiguous instructions, even in the case of machine learning. Also, currently, most end-users have no way of understanding the rules that inform the systems they use [8]. That is why we may need LPbD to safeguard (1) the democratic legitimacy of our societal infrastructure and (2) the contestability of its choice architecture.

LPbD, then, requires that the legal conditions we have agreed upon (articulated in natural language) are translated into the technical requirements that inform the data-driven architecture of our everyday environment. These requirements should instigate technical specifications and default settings that—other than current systems—afford the protection of fundamental rights. Thus, LPbD should constrain the data-driven architectures that run our new onli*f*e world, while challenging developers to offer multiple ways of modelling the world, thus making their calculations, predictions and pre-emptions both *testable* and *contestable*. Instead of ‘anything goes’ for the architects of this new world, democratically legitimated law must regain its monopoly on setting the defaults of societal order, defining the rules of the game in a way that brings the data-driven machinery under the Rule of Law.

## Saved by Design: Affordances and Capabilities

Though nanotechnology is not equivalent with the rise of a *data*-*driven environment*, this discussion note may nevertheless be pivotal for the idea of ‘safer by design’ when it comes to the reconstruction of our *physical environment*, which nanotechnology enables. This reconstruction is potentially as disruptive as cyber-physical architectures, therefore similarly requiring interventions at the level of the design of nanotechnology. To make the comparison more relevant and interesting, I will probe the notion of ‘by design’ by taking the dual perspectives of affordances and capabilities, based on James Gibson’s and Amartya Sen’s work.

Gibson developed an ecological psychology that highlights the interdependencies between an organism and its environment [9]. He framed this in terms of what an environment affords a specific organism, allowing it to flourish in its cognitive niche. He highlights the complementarity of the organism and its environment, by speaking of what the environment offers the observing organism—which obviously depends on what it is capable of perceiving and acting upon. An affordance can be positive (enabling) or negative (constraining) and is neither an objective fact (an affordance is agent-dependent) nor subjective (an affordance is contingent upon what the environment allows or constrains). It is also neither physical nor psychical, but both; an affordance is all about a perceiving and acting agent and an environment that enables such enaction,^3^ which is a matter of matter, structure, fitness and malleability: ‘an affordance points both ways, to the environment and to the observer’ ([9] 129). The notion of a cognitive niche was developed by others, underlining that interactions between an agent and its environment depend on the agent’s specific ability to *cognize* the environment, while highlighting the transformations that may result from the interaction, notably also the transformations of environment [11]. A cognitive niche, therefore, is not merely the contingent habitat of the agent, but a co-creation of both the agent and its cognitive niche. Gibson’s point is not to remain stuck in affordances that are easily perceived [12],^4^ but also to detect affordances that operate below the radar, in the back end of our immediate surroundings, setting the stage for what shows up frontstage. His relational and ecological understanding of cognition provides key conceptual tools to describe how affordances and their concomitant choice architectures change whenever the environment changes. In essence, the concept of an affordance contributes to describing how such transformations change *who and what we are*.

Sen developed an economic theory that moves beyond the opportunities to the capabilities that individuals have, which depends not only on their environment but also on the skills they develop to interact with the opportunities the environment offers them [13]. He framed capabilities in terms of our effective freedom to choose a set of valuable ‘functionings’ (‘beings and doings’), showing how a relational understanding of individual well-being helps to move beyond standard misconceptions of economic welfare. The latter tend to define welfare in terms of access to commodities, missing out on whether an individual had the chance to develop the skills to interact with such commodities in ways that allow her to enjoy her version of the good life.

Let us now combine Gibson’s relational concept of an affordance, which highlights that the affordances of an environment are agent-dependent, with Sen’s relational concept of a capability, which highlights that capabilities depend on whether an individual has learned how to make the most of what the environment affords. As should be clear, affordances and human capabilities are two sides of the same coin. A proper understanding of the dependencies between affordances (environment) and capabilities (human agent) will allow us to figure out how transformations in the technological landscape will enhance or diminish the capabilities of those living within its ambit, while this may also explain what it would mean to ‘save human capabilities by design’.

One of Kranzberg’s famous ‘laws’ states that ‘technologies are neither good nor bad, but never neutral’ [14]. This finding is built on the insight that technologies enable, enforce, inhibit or preclude specific behaviour.^5^ In terms of Gibson, one could say that new technologies will have both positive and negative affordances that, in terms of Sen, may transform human capabilities, also depending on how humans learn to interact with such technologies. The design, in the sense of *the way that specific technologies*, such as nanotechnologies, *are engineered, introduced and taken up in human society*, will make all the difference for the affordances they offer. To the extent that nanotechnologies generate new affordances, their employment will affect the capabilities of those that are forced, persuaded or invited to interact with them, or even come to depend on them. Often, the distribution of such affordances is not equivalent across different groups and individuals, depending on their ability to understand and act upon these affordances. In that sense, architecture is indeed politics [16, 17]. Whether we are speaking of physical or computational architectures, the constraints and enablers that come with specific architectures determine how knowledge, skills and power are distributed. They may, on top of that, also determine whether people have the possibility to change this distribution.

## Finals

Synthetic biology, data-driven applications, 3D printing and GMOs have new or even novel affordances. So does nanotechnology. Though these affordances are not necessarily moral or political in the narrow sense, they may redistribute or annihilate private interests and public goods such as physical safety and non-discrimination (since risks and benefits are seldom distributed evenly across a population). In the case of nanotechnology, the primary concern will be with safety, security and with uncertainty about unstable or unknown affordances. This means that we, as a society, as a democratic polity that owes equal respect and concern to each individual citizen [18], must get our act together and develop legal incentives similar to that of data protection by design (which includes security by design) for the development, marketing and employment of products that involve nanotechnology. I would argue that ‘safer by design’, however, is not a panacea and will only be effective if its implementation is required by law—attributing liability to those not interested in investing in making their product and services *safer by design*. In that sense, even ‘safer by design’ is in dire need of ‘legal protection by design’, thus turning it from an ethical choice into a legal requirement. This would bring nanotechnology under the Rule of Law [19].^6^

## Footnotes

1 Mundane notions such as ‘compliance’ or ‘nudging’ should not be confused with the force of law which is situated on the cusp of the need for legal certainty (the positivity of modern law) and the open texture of natural language (enabling and requiring interpretation, hesitation and argumentation).

2 On communication as a successful misunderstanding ([7] 28).

3 On the notion of enaction as the coupling of action and perception, where perception singles out the action potential of the environment for a specific organism, see [10].

4 Norman is focused on the visible or detectable affordances.

5 The most ingenious description of the *normative* implications of technological artefacts and infrastructures comes from Don Ihde, e.g. [15].

6 Like Waldron, I use the capital R and L to distinguish the rule of a particular law from the system of checks and balances that informs the Rule of Law.
